# Correction to “Clinical Image: Scleral melt in granulomatosis with polyangiitis”

**DOI:** 10.1002/acr2.11747

**Published:** 2024-09-17

Kaymakci MS , Elfishawi MM , Koster MJ , et al. Clinical Image: Scleral melt in granulomatosis with polyangiitis. ACROpen Rheumatol. 2023;6(1):3. doi: 10.1002/acr2.11607 PMC1078929737652888

It has come to our attention that Ashlie A. Bernhisel, MD, was mistakenly omitted as a co‐author of the aforementioned paper. She should have appeared as the second author.

Dr. Bernhisel was significantly involved in the care of the patient that is presented in this clinical image. Additionally, we acknowledge the Mayo Clinic's Department of Ophthalmology.

We apologize for this error.

